# Hypoxia negatively affects senescence in osteoclasts and delays osteoclastogenesis

**DOI:** 10.1002/jcp.26511

**Published:** 2018-06-22

**Authors:** Ben Gorissen, Alain de Bruin, Alberto Miranda‐Bedate, Nicoline Korthagen, Claudia Wolschrijn, Teun J. de Vries, René van Weeren, Marianna A. Tryfonidou

**Affiliations:** ^1^ Department of Pathobiology, Anatomy and Physiology Division, Faculty of Veterinary Medicine Utrecht University Utrecht The Netherlands; ^2^ Dutch Molecular Pathology Centre, Department of Pathobiology, Faculty of Veterinary Medicine Utrecht University Utrecht The Netherlands; ^3^ Department of Clinical Sciences of Companion Animals, Faculty of Veterinary Medicine Utrecht University Utrecht The Netherlands; ^4^ Department of Equine Sciences, Faculty of Veterinary Medicine Utrecht University Utrecht The Netherlands; ^5^ Department of Orthopedics University Medical Center Utrecht Utrecht The Netherlands; ^6^ Department of Periodontology, Academic Centre for Dentistry Amsterdam Amsterdam The Netherlands

**Keywords:** cellular senescence, hypoxia, osteoclastogenesis, osteoclasts

## Abstract

Cellular senescence, that is, the withdrawal from the cell cycle, combined with the acquirement of the senescence associated secretory phenotype has important roles during health and disease and is essential for tissue remodeling during embryonic development. Osteoclasts are multinucleated cells, responsible for bone resorption, and cell cycle arrest during osteoclastogenesis is well recognized. Therefore, the aim of this study was to investigate whether these cells should be considered senescent and to assess the influence of hypoxia on their potential senescence status. Osteoclastogenesis and bone resorption capacity of osteoclasts, cultured from CD14+ monocytes, were evaluated in two oxygen concentrations, normoxia (21% O_2_) and hypoxia (5% O_2_). Osteoclasts were profiled by using specific staining for proliferation and senescence markers, qPCR of a number of osteoclast and senescence‐related genes and a bone resorption assay. Results show that during in vitro osteoclastogenesis, osteoclasts heterogeneously obtain a senescent phenotype. Furthermore, osteoclastogenesis was delayed at hypoxic compared to normoxic conditions, without negatively affecting the bone resorption capacity. It is concluded that osteoclasts can be considered senescent, although senescence is not uniformly present in the osteoclast population. Hypoxia negatively affects the expression of some senescence markers. Based on the direct relationship between senescence and osteoclastogenesis, it is tempting to hypothesize that contents of the so‐called senescence associated secretory phenotype (SASP) not only play a functional role in matrix resorption, but also may regulate osteoclastogenesis.

## 1 INTRODUCTION

The term cellular senescence was introduced in the early 1960's (Hayflick & Moorhead, 1961), based on the observation that normal human fibroblasts stopped proliferating over time, which was speculated to be the underlying cause of aging. Currently, the process of cellular senescence is recognized as having important roles in both health and disease. It is often linked to tumor suppression, as cell cycle exiting after malignant transformation prevents tumor growth (Collado & Serrano, 2010), but more recently senescent cells have also been identified during embryological development in mammals and birds (Muñoz‐Espín et al., 2013; Nacher et al., 2006; Storer & Keyes, 2014; Storer et al., 2013). Developmental senescence, which is independent of DNA damage and dependent on the cyclin‐dependent kinase inhibitor p21, leads to the recruitment of macrophages to clear the embryo from the senescent cells (Muñoz‐Espín et al., 2013). Hence, it is essential in tissue remodeling during embryonic development.

Cyclin‐dependent kinase inhibitors, like p16 and p21 are well‐known markers of senescent cells; they indicate cell cycle exit (Muñoz‐Espín & Serrano, 2014). Their presence often coincides with the absence of Ki67, which is present in actively proliferating cells (during G1, S, G2, and M phases of the cell cycle), but absent in resting (G0 phase) cells (Gerdes et al., 1984; Scholzen & Gerdes, 2000). Exit from the cell cycle is also a feature of quiescent cells (Terzi, Izmirli, & Gogebakan, 2016). In contrast to the quiescent state, senescent cells acquire the so‐called senescence associated secretory phenotype (SASP) (Terzi et al., 2016), characterized by the production and secretion of soluble signaling factors (Coppé, Desprez, Krtolica, & Campisi, 2010). The most widely used histological method to differentiate between quiescent and senescent cells is positivity for the senescence associated beta‐galactosidase staining (Dimri et al., 1995; Itahana, Campisi, & Dimri, 2007).

Cellular senescence is also observed in healthy adult individuals and considered to be physiological. Both megakaryocytes, formed by endomitosis without cytokinesis (Besancenot et al., 2010) and placental syncytiotrophoblasts, formed by fusion of cytotrophoblasts (Chuprin et al., 2013) become senescent during their development. Senescence of these cells is speculated to play an essential, yet largely unexplored, role in their specific function while it limits oncogenic transformation.

Osteoclasts contain multiple nuclei as well. They are formed by fusion from monocyte precursor cells under the influence of M‐CSF (Dobbins et al., 2002; Van Wesenbeeck et al., 2002) and RANK‐L (Lacey et al., 1998; Wong et al., 1997; Yasuda et al., 1998). A fairly recent concept of osteoclasts is that they can renew themselves by fusion of new mononuclear precursor cells, by splitting off multinucleated daughter cells (fission) or even by fusion of existing multinucleated cells (Jansen, Vermeer, Bloemen, Stap, & Everts, 2012). They show several characteristics of senescence, such as being beta galactosidase positive at pH 7.0–8.0 (Kopp, Hooper, Shmelkov, & Rafii, 2007) but also at pH 6.0 (Chen et al., 2007), which is a hallmark of senescent cells (Dimri et al., 1995). The necessity of cycle arrest during osteoclastogenesis is well recognized (Kwak et al., 2005; Kwon et al., 2016; Mizoguchi et al., 2009; Sankar, Patel, Rosol, & Ostrowski, 2004; Takahashi, Muto, Arai, & Mizoguchi, 2010; Zauli et al., 2007) and indeed several different cyclin dependent kinase (CDK) inhibitors, among others p21, p27 and p38, are reported to be expressed during osteoclast differentiation (Chen et al., 2007; Cong et al., 2017; Okahashi et al., 2001). Further, they secrete hydrochloric acid and proteases, among others cathepsin K and matrix metallopeptidase 9 (MMP‐9) (Odgren, Witwicka, & Reyes‐Gutierrez, 2016) that can degrade bone, which are also found in the SASP (Coppé et al., 2010). However, only Chen et al. (2007) regarded osteoclasts as senescent cells, whereas others described them as being quiescent (Kwak et al., 2005; Kwon et al., 2016; Mizoguchi et al., 2009; Sankar et al., 2004; Takahashi et al., 2010; Zauli et al., 2007), leaving the exact classification of these cells and the role of senescence in their functioning open to debate.

Osteoclasts are closely associated with vessels and play an important role during embryonic and post‐natal skeletal development, as well as in pathologic conditions of the skeleton such as periodontitis (Hienz, Paliwal, & Ivanovski, 2015) and rheumatoid arthritis (Harre & Schett, 2017). In all activities hypoxia plays an important role: the hypoxia inducible transcription factor (HIF) stimulates angiogenesis and new bone formation (Shomento et al., 2010; Wang et al., 2015). Furthermore, hypoxia and more importantly, subsequent reoxygenation have a stimulating effect on the differentiation and bone resorbing capacity of osteoclasts (Arnett et al., 2003; Fukuoka, Aoyama, Miyazawa, Asai, & Goto, 2005; Knowles, 2015), possibly mediated by HIF‐1α. Mechanistically, hypoxia and reoxygenation activate nuclear factor kappa‐light‐chain‐enhancer of activated B cells (NF‐κB) (Rupec & Baeuerle, 1995) and the production of reactive oxygen species (Granger & Kvietys, 2015), which are both linked to the induction of senescence and the SASP (Acosta et al., 2013; Hubackova, Krejcikova, Bartek, & Hodny, 2012; Nelson et al., 2012). Reoxygenation is essential, as culturing osteoclasts under constant hypoxia led to extensive cell death and dramatically reduced numbers of osteoclasts (Knowles & Athanasou, 2009). However, it remains undetermined how oxygen tension and potential senescence are related and how these factors may affect osteoclast function.

The aim of this study was to investigate the profile of osteoclasts to answer the question whether these cells should be considered senescent or not and to assess the influence of hypoxia on osteoclast function and senescence status. We hypothesized that functional osteoclasts have a senescent phenotype that is stimulated by hypoxia. To verify these hypotheses, we studied osteoclastogenesis and bone resorption capacity of osteoclasts, cultured from CD14+ monocytes under the influence of M‐CSF, RANK‐L in two oxygen concentrations, that is, normoxia (21%) and hypoxia (5%). Osteoclasts were profiled by using specific staining for proliferation and senescence markers, qPCR of a number of osteoclast and senescence‐related genes and bone resorption assay.

## 2 MATERIALS AND METHODS

### 2.1 Monocyte isolation

Buffy coats from healthy donors were obtained with donor's consent from Sanquin Blood supply (Amsterdam, the Netherlands). Peripheral blood mononuclear cells (PBMCs) were isolated from the buffy coats using Ficoll–Paque density centrifugation (Ficoll–Paque PLUS, GE Healthcare). Monocytes were positively selected by magnetic‐activated cell sorting (MACS) with anti‐CD14 labeled microbeads (Miltenyi Biotec, Cat# 130‐050‐201, RRID:AB_2665482) according to the manufacturer's instructions, using an autoMACS (Miltenyi Biotec). Purity of the isolated monocyte population was confirmed using flow cytometry on a FACSCanto II cytometer (Becton Dickinson) after incubation with a monoclonal mouse anti‐human CD45‐FITC/CD14‐PE dual‐tag antibody (Beckman Coulter, Cat# 6603909, RRID:AB_2665483). Purity was on average >90%.

### 2.2 Osteoclastogenesis

Isolated CD14+ monocytes were either seeded on glass in Nunc® Lab‐Tek® II 8 wells Chamber Slides (Sigma–Aldrich, St. Louis, MO) or in plastic 96‐wells tissue culture plates (Cellstar, Greiner Bio‐One) at a cell density of 3.0 × 10^6^ cells per cm^2^, corresponding with a cell concentration of 2.2 × 10^6^ cells per chamber and 1.0 × 10^6^ cells per well (for quantitative PCR and Western blotting). For the bone resorption assay, 1 × 10^5^ monocytes per well were seeded on top of bovine cortical bone chips in 96‐well plates according to Limonard et al. (2016). The culture medium consisted of αMEM (Thermo Fisher Scientific, Waltham, MA) supplemented with 10% fetal bovine serum (16000‐044; Gibco, Thermo Fisher Scientific), 1% Penicillin/Streptomycin (P11‐010; GE Healthcare Life Sciences), 1% Fungizone (15290; Invitrogen, Carlsbad, CA), 10 ng/ml of macrophage colony stimulating factor (M‐CSF, R&D Systems) and recombinant human nuclear factor kappa B ligand (RANK‐L, Peprotech) dissolved in 0.1% human serum albumin in PBS (HSA/PBS). Cell cultures were maintained at 37 °C, 5% CO_2_ and 5% or 21% of O_2_ and media were changed twice weekly. In total three experiments were performed, each making use of three different donors and cells cultured at 5% or 21% of O_2_. Cells were harvested after 1 day (*n* = 3), 1 week (*n* = 6), 2 weeks (*n* = 6), and 3weeks (*n* = 3) of culturing (Supplementary Table S1).

### 2.3 Immunocytochemistry

One, seven, fourteen, and twenty‐one days cultured cells were fixated at room temperature for 10 min in 4% paraformaldehyde in PBS. After washing with PBS, cells were incubated with PBS containing 0.2% Triton X–100 (Sigma–Aldrich) followed by washing in PBS. Thereafter, an incubation with 10% normal goat serum in PBS was performed to reduce background staining followed by incubation with the different primary antibodies: Monoclonal rabbit anti Ki‐67 (13.3 μg/ml; Thermo Fisher Scientific Cat# RM‐9106‐S0 RRID:AB_2341197), anti p21 (2.5 μg/ml; Santa Cruz Biotechnology, Dallas, TX, Cat# sc‐471 RRID:AB_632123) and anti p16 (6.7 μg/ml; Santa Cruz Biotechnology Cat# sc‐1207 RRID:AB_632106). Goat anti rabbit\biotin (4 μg/ml; Vector Laboratories, Burlingame, CA, Cat# BA‐1000 RRID:AB_2313606) in PBS containing 5% normal goat serum was employed as a secondary antibody. Antibody binding was made visible using 3,3′‐diaminobenzidine (DAB; Dako) and nuclei were counter‐stained with hematoxylin (H3404, Vector). Subsequently, TRAP staining was performed according to the manufacturer's instruction using a commercially available kit (Leukocyte Acid Phosphatase Staining Kit, Sigma–Aldrich). Staining for senescence was performed at 7 and 14 days by incubating the cells overnight in freshly prepared senescence associated beta galactosidase staining solution at 37 °C, according to the protocol of Dimri et al. (1995). Digital images were obtained using an Olympus BX‐60 microscope, equipped with a Leica DFC450C camera and LAS 4.7 software. For each time point, oxygen concentration, donor and staining, two chamber slides were analyzed by counting and categorizing all cells present in four standardized sites of the chamber.

### 2.4 Quantitative PCR (qPCR)

After 1, 7, 14, and 21 days of culture, cells were harvested for RT‐qPCR analysis. RNA was extracted using a commercial spin‐column kit (RNeasy Micro Kit, Qiagen, Hilden, Germany) according to the instructions of the manufacturer. RNA was quantified using a Nanodrop ND‐1000 spectrophotometer (Thermo Scientific). cDNA was made using the iScript cDNA synthesis kit with similar RNA input for all the samples. RT‐qPCR reactions were performed using Sybr Green Master mix (Thermo Fisher Scientific) for a total reaction volume of 10 μl. The primers for genes related to the osteoclast phenotype and function and senescence related genes are listed in Supplementary Table S2. These primers were validated by a gradient PCR (for retrieving the melting temperature), followed by sequencing of the amplicon (Supplementary Table S2). Ct values were normalized with four reference genes (HMBS, B2M, GAPDH, and HPRT), corrected for the RT‐qPCR efficiency and further relativized with the *norm*‐*first* method. The stability of the reference genes was assessed by the use of NormFinder (Andersen, Jensen, & Ørntoft, 2004).

### 2.5 Bone resorption

Cells were cultured for 3 weeks on bovine cortical bone chips in the presence of M‐CSF and RANKL. Thereafter, cells attached to the bone were fixated at room temperature for 10 min in 4% paraformaldehyde in PBS. Non‐specific background staining was blocked with 20% normal goat serum (Vector Laboratories) for 60 min.

After washing with PBS, bone chips were incubated with Alexa Fluor 647‐labeled bisphosphonates (Coxon, Thompson, Roelofs, Ebetino, & Rogers, 2008; Thompson, Rogers, Coxon, & Crockett, 2006) for 1 hr at room temperature, followed by washing. Nuclei were visualized with propidium iodide (Sigma–Aldrich). Bone slices were stored at 4 °C in PBS until they were analyzed by confocal laser scanning microscopy (Leica SPE‐II DMI‐4000, Leica Microsystems), using a 10× ACS APO (NA 0.3) objective at a pixel size of 733 nm (zoom 1.5, 1024 × 1024 image size) and a quadruple dichroic filter that does not reflect the excitation lines in the detector path. 3D datasets were compiled from 11 slices, spaced in Z by 5 μm. Bisphosphonate fluorescence was recorded using the 635 nm laser line and emission was detected over the 646–706 nm range using the spectral detector. Buffer control staining was performed to determine background. Propidium Iodide signal was recorded using the 561 nm laser over an emission range of 571–635 nm. Quantification of the Bisphosphonate fluorescence was performed using the surface object wizard of Imaris (version 8.2.0 RRID:SCR_007370). Representative images of the different groups are shown with minor linear intensity adjustments (Red 20/190, Blue 15/230).

### 2.6 Semi‐quantitative HIF‐1α analysis by Western blot

In order to investigate whether the cells experienced hypoxia, HIF‐1α protein expression was quantified. After washing with cold PBS, cell contents were harvested by scraping the 96‐well plates with RIPA buffer containing 0.06 nM phenylmethylsulphonyl fluoride, 17 μg/ml aprotin and 1 mM sodium orthovanadate (Sigma–Aldrich). Cells were lysed on ice for 20 min, to prevent HIF‐1α degradation followed by centrifugation for 10 min at 12,000*g*. The supernatant was stored at −20 °C until analysis. Proteins were fractionated by electrophoresis using a 7.5% acrylamide gel (Bio‐Rad Laboratories, Hercules, CA) and electro‐blotted on a Nitrocellulose Membrane (9004‐70‐0, Bio‐Rad Laboratories). After blocking with milk powder dissolved in PBS containing 1% Tween (TBS‐T 1%) for 1  hr, the blots were incubated overnight at 4 °C with the primary antibody against HIF‐1α (1.0 μg/ml; BD Biosciences Cat# 610959 RRID:AB_398272) in TBS‐T 1%. After washing with TBS‐T 1%, the blots were incubated with an HRP conjugated secondary anti‐rabbit antibody (0.33 μg/ml; Cell Signaling Technology Cat# 7074 RRID:AB_2099233) for an hour at room temperature. After obtaining the results for HIF‐1α, the blots were stripped (strip buffer containing Glycin and SDS, pH 2.0) and incubated following the same protocol as described above with a primary antibody against γ tubuline (1.0 μg/ml; Sigma–Aldrich Cat# T6557 RRID:AB_477584) to serve as a loading reference. The protein expression was visualized using ECL (GE Healthcare) in a ChemiDoc XRS System (Bio‐Rad Laboratories). Images were obtained and densities were quantified by using Image Lab software (Bio‐Rad Laboratories RRID:SCR_014210).

### 2.7 Statistical analysis

Data were analyzed using R Studio Statistical software version 3.1.2 (RRID:SCR_001905). The *p* value threshold was set at 0.05 and a correction for multiple comparisons was done with the False Discovery Rate method of Benjamini and Hochberg (1995). For the RT‐qPCR and cell counting analysis, normality of the data distribution was checked graphically and with a non‐parametric bootstrapped Shapiro–Wilks test. Asthe RT‐qPCR data were not normally distributed and the sample size was low (*n* = 3), differences between the groups were assessed by a non‐parametric bootstrapped permutation test without replacement. The number of permutations for each experimental group and gene was set at 1000 and the differences between the mean values of bootstrapped ΔCt were assessed.

As the cell counting data were not normally distributed, differences between the groups were assessed by a Cox proportional hazard model (coxph), considering donor and the different experiments as random effects. Unless indicated, results are presented as mean ± standard deviation (SD). Confidence Intervals (C.I.) were set at 95%. Effect sizes (ES) were retrieved in all cases as the non‐parametric Cliff's delta (Cliff, 1993), after bootstrapping 1000 times by a Monte Carlo simulation.

The interpretation for the present work is the following: <0.11, very small or no effect; 0.11–0.28, small effect size; 0.29–0.43, medium effect size; and >0.43, large effect size. Differences were considered as (biologically) relevant if a p value < 0.05 was found and the effect size was medium or large.

## 3 RESULTS

### 3.1 Hypoxia delays cellular fusion and with that osteoclastogenesis

**Figure 1:**
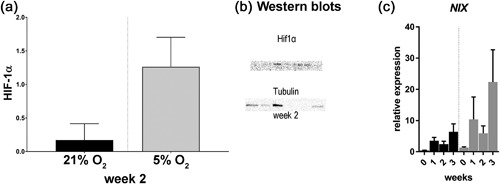
Osteoclasts seem to experience hypoxia as there is a tendency toward increased average quantity (+/− SD) of HIF‐1α present at week 2 in 5% versus 21% O_2_ (a, *n* = 3 donors per time point) and corresponding blots of HIF‐1a (upper) and tubulin (lower) (c) qRT‐PCR results of the HIF target gene NIX (BCL2 Interacting Protein 3 Like) over time (c, *n* = 3 donors per time point). There were no significant differences between culture conditions

Osteoclastogenesis was studied in CD14+ monocytes cultured in the presence of M‐CSF, RANK‐L and two oxygen concentrations, that is, normoxia (21%) and hypoxia (5%) by determining the number of multinucleated TRAP positive cells representative of an osteoclast, gene expression profiling of OCL phenotypic markers and the respective bone resorbing capacity. To confirm that cells experienced hypoxia, gene and protein expression of markers of hypoxia were determined. There were no detectable differences in the relative mRNA expression of the HIF target gene *NIX* over time between the culture conditions. At protein level HIF‐1α tended to be increased in 5% O_2_ compared to 21% O_2_ based on the *p* value of 0.090 (corrected for multiple testing) and medium effect size (Cliff's delta 0.361) (Figure 1).

**Figure 2:**
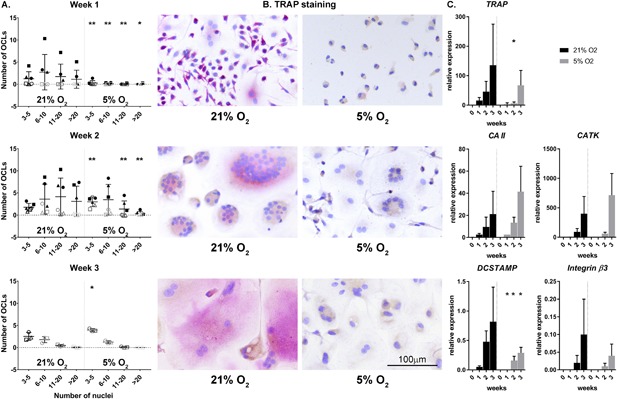
Hypoxia delays cellular fusion and with that osteoclastogenesis as indicated by the number of multinucleated, TRAP positive osteoclasts (OCL) over time (a) with representative examples (b), and corresponding results of the RT‐qPCR analysis of genes associated with osteoclast differentiation and function (c). TRAP, Tartrate‐resistant acid phosphatase, CA II, Carbonic Anhydrase II, CATK, Cathepsin K, DCSTAMP, Dendritic Cells (DC)‐Specific Transmembrane Protein, Integrin β3, Integrin subunit β3. Each symbol represents a single donor. *0.01 < *p* < 0.05; **p < 0.01 hypoxia vs normoxia at the same time point

After 1 week, significantly less osteoclasts were formed at 5% O_2_ compared to 21% O_2_. After 2 weeks, significantly more small osteoclasts (3–5 nuclei) were present at 5% O_2_, whilst significantly larger osteoclasts (>11 nuclei) were present at 21% O_2_. No significant differences were observed in the osteoclast group with 6–10 nuclei. After 3 weeks, significantly more small osteoclasts were present at 5% O_2_ (Figure 2A). TRAP staining was less intense in the osteoclasts cultured at 5% O_2_ compared to 21% O_2_ (Figure 2b). RT‐qPCR analysis revealed no significant differences in the relative expression of *Carbonic Anhydrase II*, *Cathepsin K*, and *Integrin β3* between culture conditions. The relative expression of *TRAP* was significantly lower at week 2 in the presence of 5% O_2_ compared to 21%. *DCSTAMP* was lower expressed at 5% O_2_ compared to 21% O_2_ regardless of time (Figure 2c).

**Figure 3:**
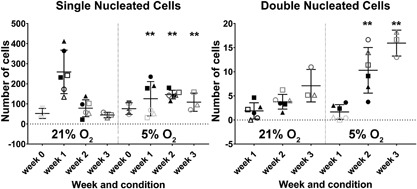
Number of single (left) and double nucleated cells after over time in normoxic (21% O_2_) and hypoxic (5% O_2_) culture conditions 21% (*0.01 < *p* < 0.05; **p < 0.01)

**Figure 4:**
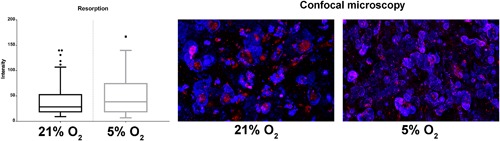
Oxygen concentration during culture does not seem to affect the bone resorption capacity of osteoclasts as indicated by the integrated intensity of bisphosphonate staining after 3 weeks of culturing osteoclasts on bovine bone chips in 21% and 5% of O_2_ (a). Representative examples with resorption lacuna stained blue and nuclei stained red (b). There were no significant differences between culture conditions

The lower number of (large) osteoclasts in the presence of hypoxia may be related either to decreased survival/adhesion of the cells at the initiation of the culture or indeed to disturbed fusion of the mononucleated cells. Therefore, the single and double nucleated cells were counted as well. No significant difference in number of single nucleated cells was observed after 1 day of culturing under normoxia or hypoxia. This indicates that at the initiation of the experiment similar numbers of mononucleated cells were present in both oxygen culture conditions. Nonetheless, significantly less single nucleated cells were present after 1 week of culturing under hypoxia, while after 2 and 3 weeks culturing the numbers of single nucleated cells were significantly higher at hypoxia compared to normoxia (Figure 3, left panel). In line with this observation, after 2 and 3 weeks of culturing under hypoxic conditions, significantly more double nucleated cells were also present (Figure 3, right panel). Altogether, these results support further the notion that the fusion of precursor cells and with that osteoclastogenesis was delayed in the presence of hypoxia. Despite the differences in OCL numbers and gene expression profile between 21% and 5% O_2_, there were no significant differences observed in bone resorption after 3 weeks of culturing, as quantified by the integrated intensity of bisfosfonate (Figure 4, left panel).

### 3.2 Osteoclasts heterogeneously express both markers of proliferation and senescence, while hypoxia seems to negatively affect senescence

**Figure 5:**
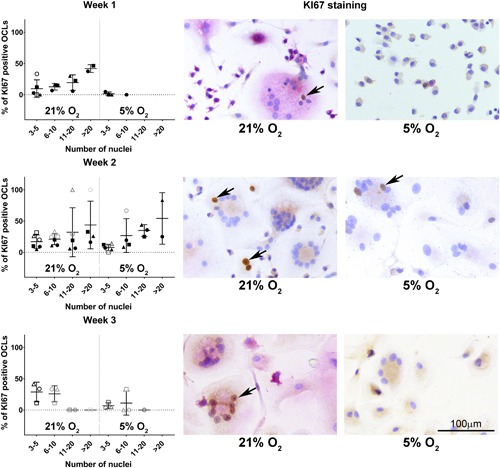
The proliferative marker Ki67 is expressed heterogeneously in the nuclei during osteoclastogenesis and seems not to be affected by oxygen tension. Percentage of Ki67 positive, multinucleated, TRAP positive osteoclasts (OCL) over time (left) with representative examples (right) in normoxic (21% O_2_) and hypoxic (5% O_2_) culture conditions. The arrows indicate Ki67 positive nuclei in multinucleated cells. There were no significant differences between culture conditions

**Figure 6:**
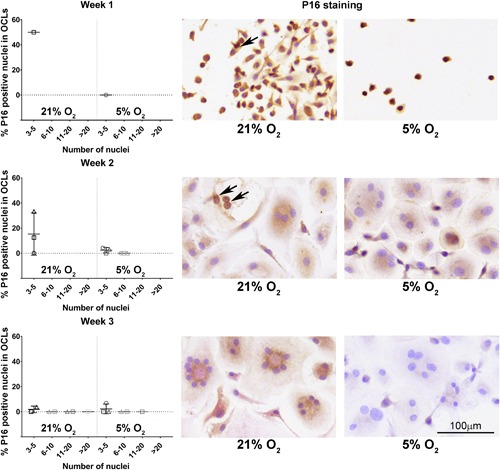
The senescence marker p16 is limited expressed during osteoclastogenesis. Percentage of p16 positive nuclei in multinucleated, TRAP positive osteoclasts (OCL) over time (left) and representative examples (right) in normoxic (21% O_2_) and hypoxic (5% O_2_) culture conditions. The arrows indicate p16 positive nuclei in multinucleated cells. Note that while mononuclear cells were p16 positive, no osteoclasts were present after 1 week of culture. There were no significant differences between culture conditions

**Figure 7:**
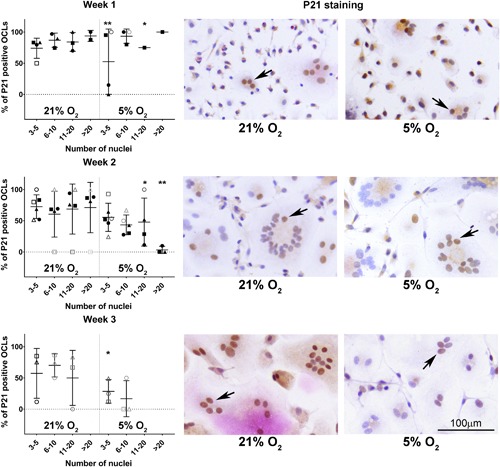
The senescence marker p21 is abundantly expressed during osteoclastogenesis and negatively affected by hypoxia. Percentage of p21 positive nuclei in multinucleated, TRAP positive osteoclasts (OCL) over time and representative examples (right) in normoxic (21% O_2_) and hypoxic (5% O_2_) culture conditions. The arrows indicate p21 positive nuclei in multinucleated cells. *0.01 < *p* < 0.05; **p < 0.01 hypoxia vs normoxia at the same time point

**Figure 8:**
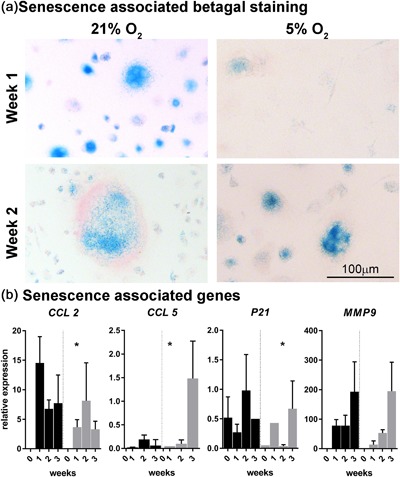
Senescence associated beta galactosidase staining is present at week 1 and 2 (a) supporting the senescent phenotype of osteoclasts. RT‐qPCR analysis of the senescence associated genes CCL 2, CCL5, p21, and MMP9 (b; *n* = 3 donors) shows differential gene expression profiles in normoxia vs hypoxia. CCL2, C‐C Motif Chemokine Ligand 2; CCL 5, C‐C Motif Chemokine Ligand 5; p21, Cyclin Dependent Kinase Inhibitor 1A; MMP9, Matrix Metalloproteinase 9 after 1 day of culturing (indicated with “0”) and 1–3 weeks of culturing. *0.01 < *p* < 0.05; **p < 0.01 hypoxia vs normoxia at the same time point

During osteoclastogenesis in two oxygen concentrations, that is, normoxia (21%) and hypoxia (5%), osteoclasts were profiled by using specific staining for proliferation and senescence markers and senescence‐related genes expression analysis. Surprisingly, osteoclasts expressed Ki67; much heterogeneity existed in the proportion of positive nuclei within osteoclasts (Figure 5). In some, all nuclei stained positive, whereas in others only one or two did so. There were no significant differences between osteoclasts cultured at hypoxia or normoxia (Figure 5). While mononuclear cells stained positive for both nuclear and cytoplasmic p16, only a small fraction of the osteoclasts showed a positive nuclear staining for p16; no significant differences were observed between cells cultured under normoxia and hypoxia (Figure 6). The cytoplasm seemed to stain positive for p16 for both oxygen conditions after 1 and 2 weeks of culture. Only at week 3, in osteoclasts cultured at 5% O_2_ cytoplasmic staining was consistently less intense compared to 21% O_2_ (Figure 6, third row). If an osteoclast stained positive, the majority of its nuclei were positive for p16. Furthermore, the majority of the osteoclasts showed a positive nuclear staining for p21 (Figure 7). Significantly less osteoclasts containing 3–5 and 11–20 nuclei and cultured at 5% O_2_ were positive compared to 21% O_2_ at week 1. At week 2, only larger osteoclasts (>11 nuclei) showed significantly less positive nuclei, whereas at week 3, significantly less small p21 positive (3–5 nuclei) osteoclasts were present in hypoxia compared to normoxia. Also here, variation was observed in the proportion of osteoclast nuclei that stained positive in all culture conditions and time points. Almost all multinucleated cells stained positive for senescence associated beta galactosidase, as evidenced by the presence of blue cytoplasmic precipitate, independent of O_2_ concentration during culture (Figure 8a). Comparison of the relative expression of senescence associated genes showed significantly lower expression of *CCL2* after 1 week and of *p21* after 2 weeks under hypoxia compared to normoxia, while the opposite is true for *CCL5* expression after one week. No significant differences were observed in the relative expression of *MMP9* (Figure 8b).

## 4 DISCUSSION

The results of this study suggest that during in vitro osteoclastogenesis, osteoclasts obtain a senescent phenotype and that fusion of precursor cells, an essential initial step in osteoclastogenesis was delayed in the presence of 5% O_2_ compared to 21% O_2_ without negatively affecting the bone resorption capacity of the cells on the long term.

### 4.1 Osteoclasts express a senescent phenotype

Cell cycle arrest is a hallmark of osteoclastogenesis and based hereon osteoclasts have always been described as quiescent cells (Kwak et al., 2005; Kwon et al., 2016; Mizoguchi et al., 2009; Sankar et al., 2004; Takahashi et al., 2010; Zauli et al., 2007). However, the present study indicates that they should be considered senescent rather than quiescent. Senescence is not limited to the arrest of proliferation, but also includes the acquirement of the senescence associated secretory phenotype (SASP) (Terzi et al., 2016), characterized by the production and secretion of soluble signaling factors (Coppé et al., 2010). In the current study, osteoclasts indeed expressed typical markers of senescence, including p21 and senescence associated beta galactosidase, in line with a previous report (Chen et al., 2007). In fact, the expression profile of the osteoclasts derived from PBMCs stimulated with M‐CSF and RANK‐L overlaps with the SASP. We observed increasing expressing of *CCL2* and *CCL5*, which are known to be part of the SASP and have chemotactic properties (Ruhland et al., 2016). Notably, these substances also stimulate osteoclastogenesis (Khan, Hashimi, Bakr, Forwood, & Morrison, 2016; Kim et al., 2015) and production of MMP‐9 (Chuang et al., 2009). MMP‐9 is also a part of the SASP and an important enzyme for resorbing bone (Coppé et al., 2010). The expression of *Cathepsin K*, which is an enzyme produced by mature osteoclasts to break down the non‐mineralized bone matrix, increased over time in both culturing conditions. Interestingly, besides its matrix degrading characteristics, *Cathepsin K* can also induce senescence in osteoclasts, possibly to control and limit their number (Chen et al., 2007). Altogether, these findings indeed imply that osteoclasts obtain a senescence‐associated secretory phenotype and strongly suggest that the SASP secretome exerts paracrine effects that possibly regulate osteoclastogenesis and bone resorption.

### 4.2 Oxygen tension influences osteoclast senescence

As determined by senescence and proliferation markers. Contrary to our hypothesis, oxygen concentration was inversely correlated with the percentages of positively stained p21 nuclei in osteoclasts, a marker of senescence. This indicates that senescence was negatively affected by decreased oxygen tension. Cyclin‐dependent kinase inhibitor 2A or p16 is another senescence marker that typically has very low expression in young and healthy tissue. However, as it is activated by cellular damage or stress, it is abundant in aged tissues (Krishnamurthy et al., 2006; Zindy, Quelle, Roussel, & Sherr, 1997) and therefore considered to be a good senescence marker for in vivo studies (Baker et al., 2011; Burd et al., 2013; Yamakoshi et al., 2009). In the present study, only a very limited number of nuclei were positive for p16, whereas cytoplasmic p16 was present in the majority of the cells, both in single and multinucleated ones. Within the context of osteoclastogenesis, the presence of p16 together with p21 has also been reported in murine monocytes cultured to become osteoclasts (Cong et al., 2017). Cytoplasmic p16 localization has been related to malignancy (McCluggage & Jenkins, 2003; Reid‐Nicholson et al., 2006; Zhao et al., 2012), but its biological meaning is still under debate. The fact that nuclear p21 was primarily affected in osteoclasts cultured in hypoxia, may be explained by the differences between cellular senescence in adult cells and in developing cells. Storer et al. (2013) observed that in senescent cells of developing embryos, p21 was present, while they were unable to detect p16 or DNA damage; which are both essential features in replicative and oncogene induced cellular senescence of adult cells. To our surprise, we observed that a considerable percentage of osteoclasts were positive for Ki67. The presence of Ki67 in the osteoclasts can obviously not be related to proliferation, as their cell cycle is arrested. However, Ki67 has also been shown to be present in quiescent cells, possibly associated with ribosomal RNA transcription (Bullwinkel et al., 2006; Rahmanzadeh, Hüttmann, Gerdes, & Scholzen, 2007). Another potential reason for the presence of Ki67 positive nuclei might be the possible occurrence of fission of osteoclasts (Jansen et al., 2012). We did not study this, but it has been described that multinucleated osteoclasts can split into two or more multinucleated daughter cells. This cytoplasmic separation has some resemblance with the last phases of mitosis, which might explain presence of Ki67.

### 4.3 Hypoxia delays osteoclastogenesis without long term effect on bone resorption capacity

The fact that we observed in general less large osteoclasts and more single and double cells under hypoxic culture conditions pointed at delayed osteogenesis. Furthermore, at the gene expression level, several osteoclast‐markers were markedly downregulated by hypoxia, example, *TRAP* and *DCSTAMP*. RANK‐L in presence of M‐CSF stimulates proliferation of the mononuclear osteoclast precursors. In this process, the cell density and number of cells present prior to cell fusion can influence the number of osteoclasts that eventually form (Cong et al., 2017; Motiur Rahmanet al., 2015). However, these two factors were not different between the hypoxic and normoxic culture conditions in our study and similar numbers of adherent cells were observed after 24 hr of culturing in either culture. Possibly, the lower number of mononuclear cells observed after one week is the result of the inhibiting effect of hypoxia on their proliferation (Naldini & Carraro, 1999). While delayed osteoclastogenesis seems to be in contrast to several other papers reporting positive effects of hypoxia on osteoclastogenesis (Arnett et al., 2003; Fukuoka et al., 2005; Knowles, 2015), it confirms others reporting negative effects of hypoxia on osteoclast formation (Leger et al., 2010; Hulley et al., 2017). These contradictory observations may be related to differences in culture set up and to the pH sensitivity of the medium, where even different brands of fetal calve serum could have an influence. Although 2% O_2_ has been reported to be the optimal concentration for bone resorption (Knowles, 2015), culturing osteoclasts under constant hypoxia leads to extensive cell death and dramatically reduced osteoclast numbers (Knowles & Athanasou, 2009). Therefore, we cultured at 5% O_2_ and allowed reoxygenation twice a week during culture medium change. This frequency of medium change and intermittent exposure to normoxia is comparable to the setup of Hulley et al. (2017), who also reported decreased osteoclastogenesis. Nonetheless, while osteoclastogenesis was delayed in hypoxic culture conditions, gene expression of *CAII, CATK*, and *MMP9*, secreted by osteoclasts to resorb bone did not differ between conditions and resorption capacity at week 3 was comparable in the two culture conditions.

### 4.4 The study has several limitations

Five percent O_2_ did not produce a robust down‐regulation of HIF‐1α protein expression, but did affect the senescence phenotype and osteoclastogenesis. It remains to be determined whether O_2_ concentrations lower than 5% would elicit a distinct response. Regulations at cellular level induced by long term hypoxia, including mRNA HIF‐1a stability (Uchida et al., 2004) and degradation of HIF‐1α by the proteasome (Demidenko et al., 2005) may also account for the absence of a distinct response at HIF‐1α protein level to the hypoxic stimulus. Donor background information is lacking, which could have influenced our results, as for example a clear relation with age has been shown for the presence of p16 positive staining of nuclei in healthy tissue (Krishnamurthy et al., 2006; Zindy et al., 1997). There was distinct donor variability resulting in differences in pace of osteoclastogenesis between the different experiments. For this reason, each donor was represented by a unique symbol in the figures. This limitation does not necessarily affect interpretation and generalization of the results, as each donor was employed in both culture conditions (hypoxia and normoxia). Inherent to the in vitro model and chosen set up is the discrepancy with osteoclast formation on bone that follows a different dynamic than on plastic (De Vries, Schoenmaker, Hooibrink, Leenen, & Everts, 2009). Sometimes, inhibitory effects on plastic can be nullified on bone (De Vries et al., 2015).

## 5 CONCLUSION

The present study suggests that osteoclasts can be considered senescent instead of quiescent. Notably, senescence is not uniformly present in the osteoclast population, which may represent different stages in the life of the osteoclast. This may also be the background of the heterogeneous expression of Ki67, which is indicative of augmented ribosomal RNA transcription rather than proliferative activity. The direct relationship between senescence and osteoclastogenesis might mean that contents of the SASP not only play a functional role in matrix resorption but also may regulate osteoclastogenesis in a paracrine manner. Hypoxia seems to affect the expression of senescence markers negatively and cellular fusion and formation of large osteoclasts is delayed at hypoxic (5% of O_2_) compared to normoxic conditions (21% O_2_). This, however, does not affect the resorption capacity of the osteoclasts on the longer term.

## Supporting information

Additional Supporting Information may be found online in the supporting information tab for this article.

**Table S1**. Experimental setup.

**Table S1**. Primer information.
