# Supplementary material for: Hypoxia negatively affects senescence in osteoclasts and delays osteoclastogenesis
Source: J Cell Physiol. 2018 Jun 22;234(1):414–26. doi: 10.1002/jcp.26511 (PMC6220985; doi:10.1002/jcp.26511)
Supplement: Supplementary file 1 — Table S1. Experimental setup. [file JCP-234-414-s001.docx]

**Supplementary table 1: Experimental setup**

| Technique | | Immunocytochemistry  (Nunc® Lab-Tek® II 8 wells Chamber Slides) | RT-qPCR  (plastic 96-wells tissue culture plates) | Western blot  (plastic 96-wells tissue culture plates) |
| --- | --- | --- | --- | --- |
| Cells harvested after: | Medium | Number of donors used: | | |
| 1 day | plain medium | 3 | 3 |  |
| 1 week | RANK-L & M-CSF | 6 | 3 |  |
| 2 weeks | RANK-L & M-CSF | 6 | 3 | 3 |
| 3 weeks | RANK-L & M-CSF | 3 | 3 |  |

*Supplementary table 1: Setup of all experiments and number of different donors used for each of the read out parameters.*
