# Supplementary material for: Hypoxia negatively affects senescence in osteoclasts and delays osteoclastogenesis
Source: J Cell Physiol. 2018 Jun 22;234(1):414–26. doi: 10.1002/jcp.26511 (PMC6220985; doi:10.1002/jcp.26511)
Supplement: Supplementary file 2 — Table S1. Primer information. [file JCP-234-414-s002.docx]

**Supplementary table 2: Primer information**

| **Genes** | **Forward sequensce 5' - 3'** | **Reverse sequence 5' - 3'** | **Amplicon size** | **Annealing temp. (°C)** | **Accession no.** |
| --- | --- | --- | --- | --- | --- |
| **Reference genes** |  |  |  |  |  |
| *CDC73* | TATTGTAATGACCAGTCAACAG | GGTCCTTTTCACCAGCAAG | 192 | 60 | NM_024529.4 |
| *HMBS* | GGCAATGCGGCTGCAA | GGGTACCCACGCGAATCAC | 64 | 56 | NM_000190.3 |
| *B2M* | CTTTGTCACAGCCCAAGATAG | CAATCCAAATGCGGCATCTTC | 83 | 58 | NM_004048.2 |
| *GAPDH* | TGCACCACCAACTGCTTAGC | GGCATGGACTGTGGTCATGAG | 87 | 62 | NM_001256799.2 |
| **Target genes osteoclasts** |  |  |  |  |  |
| *CA2* | TGGACTGGCCGTTCTAGGTATT | TCTTGCCCTTTGTTTTAATGGAA | 100 | 59 | NM_000067.2 |
| *CTSK* | CCATATgTGGGACAGGAAGAGAGTT | TGCATCAATGGCCACAGAGA | 149 | 66 | NM_000396.3 |
| *ACP5* | CACAATCTGCAGTACCTGCAAGAT | CCCATAGTGGAAGCGCAGATA | 128 | 68 | NM_001111035.2 |
| *DCSTAMP* | ATTTTCTCAGTGAGCAAGCAGTTTC | AGAATCATGGATAATATCTTGAGTTCCTT | 101 | 61,5 | NM_030788.3 |
| *ITGB3* | AGGCTGGCAGGCATTGTC | AGCCCCAAAGAGGGATAATCC | 100 | 66 | NM_000212.2 |
| **Target genes senescence** |  |  |  |  |  |
| *CCL2* | GATCTCAGTGCAGAGGCTCG | TGCTTGTCCAGGTGGTCCAT | 153 | 60 | NM_002982.3 |
| *CCL5* | GCTGCTTTGCCTACATT | CATTTCTTCTCTGGGTTG | 141 | 59 | NM_002985.2 |
| *MMP9* | CCTGGAGACCTGAGAACCAATC | TTCGACTCTCCACGCATCTCT | 100 | 66 | NM_004994.2 |
| *CDKN1A* | CTCTAAGGTTGGGCAGGGTGACC | CAGAGGGGGGTATCAAGAGCCAG | 91 | 68 | NM_000389.4 |
| **Target genes hypoxia** |  |  |  |  |  |
| *BNIP3L* | AGTAGCTTATTTGAACTTGAGACCATTG | TGAGGGTTACTGGAATTGGATATGTA | 83 | 61,5 | NM_004331.2 |

*Supplementary table 2: Primer sequences*, a*mplicon size, annealing temperature (°C) and Genbank accession number of the primers used for the RT-qPCR analysis. CDC73 (HRPT1):* *cell division cycle 73,* *HMBS: hydroxymethylbilane synthase,* *B2M: beta-2-microglobulin, GAPDH:* *glyceraldehyde-3-phosphate dehydrogenase,* *CA2 (CAII): Carbonic Anhydrase II,* *CTSK (CATK): Cathepsin K, ACP5 (TRAP): Tartrate-resistant acid phosphatase, DCSTAMP: Dendritic Cells (DC)-Specific Transmembrane Protein, ITGB3:* (*Integrin β3): Integrin subunit beta 3,* *CCL2: C-C Motif Chemokine Ligand 2,* *CCL 5: C-C Motif Chemokine Ligand 5, MMP9: Matrix Metallopeptidase 9,* *CDKN1A (P21): Cyclin Dependent Kinase Inhibitor 1A,.* BNIP3L (*NIX): BCL2 Interacting Protein 3 Like.*
